# Supplementary material for: Comparative multiomics analysis of cell physiological state after culture in a basket bioreactor
Source: Sci Rep. 2022 Nov 23;12:20161. doi: 10.1038/s41598-022-24687-4 (PMC9686226; doi:10.1038/s41598-022-24687-4)
Supplement: Supplementary file 1 — Supplementary Information 1. [file 41598_2022_24687_MOESM1_ESM.zip › raw data/Transcriptomic raw data/6.Enrichment/2.KEGG/TE1vsTC1/ALL/src/csab05202.html]

csab05202


- csab:103219133

- Up regulated genes

103219133(2.88578313336763)

- csab:103237430

- Up regulated genes

103237430(1.35562492669487)

- csab:103226390

- Up regulated genes

103226390(2.68260263702081)

- csab:103228427

- Up regulated genes

103228427(1.62058418943349)

- csab:103237430

- Up regulated genes

103237430(1.35562492669487)

- csab:103218917

- Down regulated genes

103218917(-2.19478759342619)

- csab:103219133

- Up regulated genes

103219133(2.88578313336763)

- csab:103215685

- Down regulated genes

103215685(-1.98840718122177)

- csab:103224652

- Up regulated genes

103224652(1.58918989505374)

Close
